# Microfluidic rotating-target device capable of three-degrees-of-freedom motion for efficient *in situ* serial synchrotron crystallography

**DOI:** 10.1107/S1600577523000462

**Published:** 2023-02-15

**Authors:** Feng-Zhu Zhao, Zhi-Jun Wang, Qing-Jie Xiao, Li Yu, Bo Sun, Qian Hou, Liang-Liang Chen, Huan Liang, Hai Wu, Wei-Hong Guo, Jian-Hua He, Qi-Sheng Wang, Da-Chuan Yin

**Affiliations:** a School of Life Sciences, Northwestern Polytechnical University, Xi’an 710072, People’s Republic of China; b School of NCO, Army Medical University, Shijiazhuang 050081, People’s Republic of China; c Shanghai Advanced Research Institute, Chinese Academy of Sciences, Shanghai 201800, People’s Republic of China; d Shanghai Institute of Applied Physics, Chinese Academy of Sciences, Shanghai 201800, People’s Republic of China; e School of Materials Science and Engineering, Northwestern Polytechnical University, Xi’an 710072, People’s Republic of China; f The Institute for Advanced Studies, Wuhan University, Wuhan 430072, People’s Republic of China; Paul Scherrer Institute, Switzerland

**Keywords:** sample delivery system, serial synchrotron crystallography, microfluidic rotating-target, circular motion, *in situ* diffraction

## Abstract

There is an increasing demand for simple and efficient sample delivery technology to match the rapid development of serial crystallography and its wide application in analyzing the structural dynamics of biological macromolecules. Here, a microfluidic rotating-target device is presented, capable of three-degrees-of-freedom motion, including two rotational degrees of freedom and one translational degree of freedom, for sample delivery. Lysozyme crystals were used as a test model with this device to collect serial synchrotron crystallography data and the device was found to be convenient and useful. This device enables *in situ* diffraction from crystals in a microfluidic channel without the need for crystal harvesting. The circular motion ensures that the delivery speed can be adjusted over a wide range, showing its good compatibility with different light sources. Moreover, the three-degrees-of-freedom motion guarantees the full utilization of crystals. Hence, sample consumption is greatly reduced, and only 0.1 mg of protein is consumed in collecting a complete dataset.

## Abstract 2

A novel microfluidic rotating-target device capable of three-degrees-of-freedom motion has been proposed, and its feasibility for sample delivery was successfully verified at a synchrotron radiation facility. This device guarantees the full utilization of crystals and only 0.1 mg of protein is consumed in collecting a complete dataset.

## 1. Introduction

The development of serial crystallography (SX) offers new opportunities to the fields of crystallography, biology and medicine. The advantages of SX include structural determination at room temperature (Ayan *et al.*, 2022 ▸) at crystal sizes down to the micrometre or even submicrometre level. Furthermore, SX is an ideal tool for carrying out high-resolution time-resolved studies (Pearson & Mehrabi, 2020 ▸; Orville, 2020 ▸; Dods *et al.*, 2021 ▸; Pandey *et al.*, 2020 ▸), such as enzymatic reactions and light activation of proteins (Kwon *et al.*, 2021 ▸; Li *et al.*, 2021 ▸; Poddar *et al.*, 2022 ▸; Yun *et al.*, 2021 ▸; Oda *et al.*, 2021 ▸; Suga *et al.*, 2019 ▸; Wiedorn *et al.*, 2018 ▸; Olmos *et al.*, 2018 ▸). SX experiments can also be used for *de novo* phase studies (Lawrence *et al.*, 2020 ▸; Nass *et al.*, 2020 ▸; Huang *et al.*, 2018 ▸) and the structural analysis of viruses (Roedig *et al.*, 2017 ▸; Meents & Wiedorn, 2019 ▸; Lawrence *et al.*, 2015 ▸). SX experiments can be performed at room temperature thanks to the mechanism of ‘diffraction before destruction’ in serial femtosecond crystallography (SFX) (Neutze *et al.*, 2000 ▸) and the ability to spread the dose over multiple crystals in serial synchrotron crystallography (SSX) (Ebrahim *et al.*, 2019 ▸). The rise and development of SX has also promoted a renaissance of room-temperature crystallography because conformation heterogeneity related to function can be observed at room temperature but might be changed under cryogenic conditions (Fraser *et al.*, 2011 ▸; Russi *et al.*, 2017 ▸; Echelmeier *et al.*, 2020 ▸). In addition, studies have shown that global radiation damage and specific radiation damage at room temperature exhibit less decoupling than those under cryogenic conditions (Gotthard *et al.*, 2019 ▸).

Many related technologies have been developed for better SX practices, including crystal detection technology (Cheng *et al.*, 2020 ▸), *in situ* diffraction devices and sample delivery systems. The sample delivery system is one of the key links in the implementation of SX research, and it is also an important tool for diversified experiments using SX technology. Practically, the sample delivery system is also the main limiting factor affecting the application of SX and has been increasingly studied by researchers. A number of sample delivery systems which can be categorized as liquid jet technology and fixed-target technology as well as hybrid methods have been developed (Echelmeier *et al.*, 2019 ▸; Zhao *et al.*, 2019 ▸; Cheng, 2020 ▸; Grünbein & Kovacs, 2019 ▸; Echelmeier *et al.*, 2020 ▸; Ren *et al.*, 2020 ▸; Monteiro *et al.*, 2020 ▸; Li *et al.*, 2018 ▸). In addition, various novel sample delivery systems, including upgrades to existing sample delivery systems, are constantly emerging for better SX practice (Gilbile *et al.*, 2021 ▸; Nam, 2022 ▸; Martiel *et al.*, 2021 ▸; Doppler *et al.*, 2022 ▸; Lee *et al.*, 2022 ▸; Patel *et al.*, 2022 ▸; Vakili *et al.*, 2022 ▸; Hammarström *et al.*, 2022 ▸). These devices can be applied to different light sources, including X-ray free-electron laser (XFELs) and synchrotron radiation (SR) sources, or both, depending on their delivery speed. In general, XFEL sources require a higher sample delivery speed, and SR sources require a slower sample delivery speed to obtain effective diffraction data that can be used for structural analysis. In addition, experiments using XFELs with a low repetition rate also require late sample delivery.

Since there are far more SR sources than XFEL sources worldwide, the development of SSX has provided opportunities for more researchers to conduct SX experiments, thereby providing new opportunities for the widespread application of SX (Lan *et al.*, 2018 ▸). With the increase in SSX experiments, there is an increasing demand for developing sample delivery systems that demonstrate low consumption and high efficiency for SR sources; moreover, SFX experiments also have a variety of sample delivery system requirements. Therefore, the trend continues for developing sample delivery devices suitable for SR sources, XFEL sources, or both.

In our previous research, we developed a circular-motion-based sample delivery device; that is, a microfluidic rotating-target device capable of two-degrees-of-freedom motion (2-DOF-MRT), and its sample delivery performance was verified at the Shanghai Synchrotron Radiation Facility (SSRF). The sample delivery study of the 2-DOF-MRT device pioneered a new sample delivery mode based on circular motion for the first time, which was different from the existing technologies that adopted linear or multisegment linear motion for sample delivery. The circular motion feature allows the sample delivery speed to be easily adjusted over a wide range, giving the 2-DOF-MRT device the potential to be applied in many different SR facilities (Zhao *et al.*, 2020 ▸).

Inspired by oscillating serial crystallography (Guo *et al.*, 2018 ▸; Gati *et al.*, 2014 ▸; Wierman *et al.*, 2019 ▸), we believe that the crystal still has the ability to provide effective diffraction frames after a single short-term exposure of the SR source (depending on the radiation dose rate). However, in our study of the 2-DOF-MRT device, each crystal contributed at most one effective diffraction frame. If these crystals are rotated, more than one diffraction frame from the same crystal can be collected. Therefore, we propose to add a rotational degree of freedom based on the 2-DOF-MRT device, that is, a microfluidic rotating-target device capable of three-degrees-of-freedom motion (3-DOF-MRT), including two rotational degrees of freedom and one translational degree of freedom. Then, multiple effective diffraction frames can be collected from each crystal to further reduce sample consumption and achieve the goal of collecting a complete dataset from a single microfluidic sample plate.

Herein, a 3-DOF-MRT device is developed and successfully tested on beamline BL18U1 of SSRF by using lysozyme as a test model. This device combines oscillating serial crystallography and circular-motion-based serial crystallography and exhibits attractive features; for example, the circular motion ensures that the delivery speed can be adjusted over a wide range [0–20 revolutions min^−1^ (hereafter RPM) – the corresponding sample speed range is approximately 0–1.36 × 10^−2^ m s^−1^], showing its good compatibility with different light sources. The *in situ* diffraction avoids the need for crystal harvesting. The 3-DOF-MRT guarantees the full utilization of crystals. Hence, sample consumption is greatly reduced, and only 0.1 mg protein is consumed for collecting a complete dataset within 6.5 h. Finally, we discuss the selection of motion control parameters for SSX research with this device to provide a reference for its application.

## 2. Materials and methods

### 2.1. Design of the 3-DOF-MRT device

**Figure 1:**
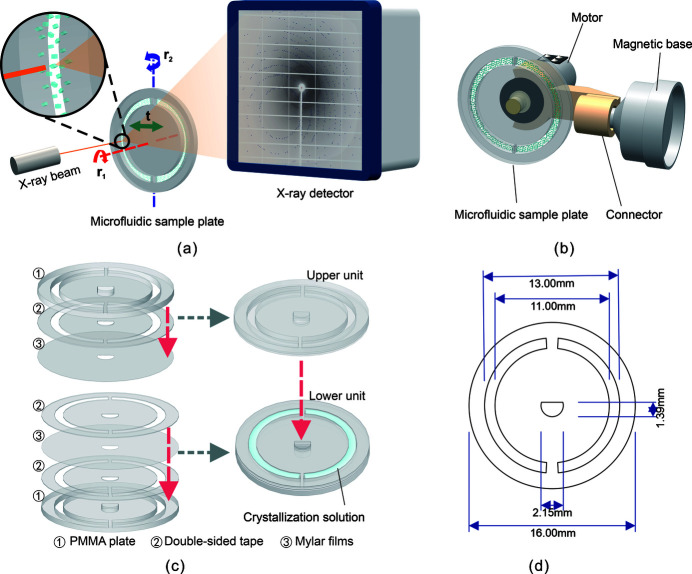
Design of the 3-DOF-MRT device. (*a*) Overall design of the 3-DOF-MRT device. *r*_1_: rotational degree of freedom; *t*: translational degree of freedom; *r*_2_: rotational degree of freedom. (*b*) Schematic diagram of the overall exterior of the 3-DOF-MRT device. (*c*) Structure and assembly process of the microfluidic sample plate. (*d*) Size of the microfluidic sample plate.

The design of the 3-DOF-MRT device involves two parts: a microfluidic sample plate and a motion control system. As shown in Fig. 1 ▸(*a*), the purpose of the 3-DOF-MRT device is to control the microfluidic sample plate containing crystals to rotate (*r*_1_ and *r*_2_) and translate (*t*) through a 3-DOF motion control system to realize efficient sample delivery. The overall exterior of the 3-DOF-MRT device is shown in Fig. 1 ▸(*b*).

#### 2.1.1. Microfluidic sample plate

The microfluidic sample plate is the carrier that drives the movement of crystals, and its structural design was embodied in the 2-DOF-MRT device (Zhao *et al.*, 2020 ▸). The important feature of the microfluidic sample plate is its disk-like geometry that is suitable for circular motion. Moreover, the microfluidic sample plate has a channel with a depth of 140 µm for carrying crystals on the micrometre scale. In fact, it is possible to manufacture smaller microfluidic channels to accommodate smaller crystals by choosing thinner double-sided tape. For example, 3M can offer products as thin as 10 µm, which means that the available crystal size can be reduced to approximately 10 µm. The microfluidic sample plate was divided into an upper unit and lower unit for assembly according to Fig. 1 ▸(*c*). The upper unit was obtained by placing double-side tape, a polymethyl methacrylate (PMMA) plate, double-sided tape and Mylar film layer-by-layer, and the lower unit was obtained by placing double-side tape, Mylar film and a PMMA plate layer-by-layer. The crystallization solution was loaded into the microfluidic channel of the lower unit by pipette, and then a complete microfluidic sample plate was assembled by affixing the upper unit to the lower unit. To adapt to the new motion control strategy, the size and appearance of the microfluidic sample plate were adjusted based on our previous study. Details of the size of the microfluidic sample plate are presented in Fig. 1 ▸(*d*).

#### 2.1.2. 3-DOF motion control system

The 3-DOF motion control of the microfluidic sample plate relies on the combination of a motor and goniometer in function and structure. The microfluidic sample plate was installed on the motor shaft through a hole machined in its middle that matched the motor shaft. We designed and machined a ‘connector’ to join the motor to the magnetic base (CrystalCap Copper Magnetic). Thus, the entire 3-DOF-MRT device could be installed in the diffraction facility through the magnetic base.

Due to space limitations, it is not possible to directly apply the rotational (*r*_2_) degree of freedom to the 2-DOF-MRT device. Moreover, the microfluidic sample plate of 2-DOF-MRT was not on the same axis as the goniometer; thus, the rotation angle resolution was not accurate when rotated by the goniometer. Therefore, in the design of the 3-DOF-MRT device, a bent ‘connector’ was designed, as shown in Fig. S1 of the supporting information, so that the microfluidic sample plate and goniometer were on the same axis to achieve precise control of the rotational (*r*_2_) degree of freedom.

**Figure 2:**
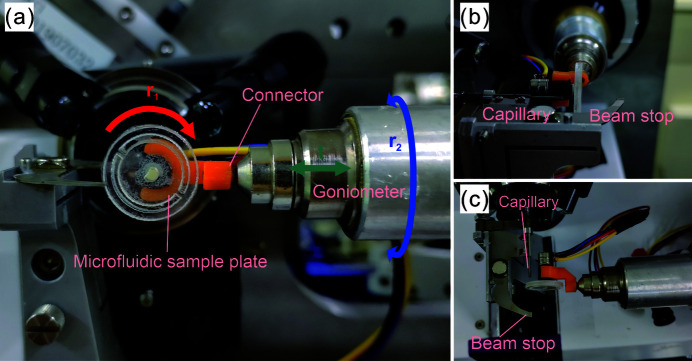
Physical images of the 3-DOF-MRT device and its status when used for the SSX experiment. (*a*) Front view of the 3-DOF-MRT device. The red arrow indicates the rotational (*r*_1_) degree of freedom, the green arrow indicates the translational degree of freedom, and the blue arrow indicates the rotational (*r*_2_) degree of freedom. (*b*) Side view of the 3-DOF-MRT device. (*c*) Top view of the 3-DOF-MRT device.

As shown in Fig. 1 ▸(*a*), the rotational (*r*_1_) degree of freedom (red arrow) was controlled by a motor. By selecting motors with different models, the rotational speed could be controlled in different ranges. The translational degree of freedom (green arrow) was controlled by the goniometer, and the goniometer was programmed to translate along the *z*-axis to realize the control of translational motion. The second rotational (*r*_2_) degree of freedom (blue arrow) was also controlled by the programmed goniometer, which controlled the rotational (*r*_2_) degree of freedom by programming the goniometer to rotate. Fig. 2 ▸ shows physical images of the 3-DOF-MRT device and its status used for the SSX experiment on BL18U1 of the SSRF. It can be seen that the designed device was completely compatible with the diffraction environment.

In this study, the rotational (*r*_1_) motion controlled by the motor was carried out at a speed of 1/6 RPM. A translation movement was performed after one circle of rotation; that is, translation occurred once every 6 min, with a translation step of 100 µm; a total of seven translations occurred. Then, the goniometer was used to reverse translate to the initial position and perform one rotational (*r*_2_) motion at an angle resolution of 1°. The above rotation and translation motion was repeated until enough diffraction data were obtained for analysis. In our experiment, a total of seven (*r*_2_) rotations were performed, and diffraction data from −4° to 3° (as determined by the goniometer) were collected. An animation demonstration of the motion control scheme is displayed in Movie S1 of the supporting information.

### 2.2. Manufacturing of the 3-DOF-MRT device

The manufacture of the 3-DOF-MRT device involved the manufacture of a microfluidic sample plate and a ‘connector’, as well as the assembly of a microfluidic sample plate and the entire device. The Mylar film, double-sided tape and a PMMA plate were chosen as the materials for the microfluidic sample plate. The Mylar film was purchased from Shanghai East Electronic Co. Ltd., and its thickness was 3.6 µm. The double-sided tape (3M9500PC) was purchased from the 3M Company, and its thickness was 140 µm, which could provide a spacer for microlevel crystal growth. The PMMA plate was purchased from Xi’an Shuguang Polymethyl Methacrylate Products Co. Ltd, and its thickness was 1 mm, providing sufficient stiffness. In addition, the PMMA plate exhibited good processability and could flexibly cope with complex processing requirements. Polylactic acid (PLA) was selected as the material for manufacturing the ‘connector’ by 3D printing. The motor (AZH1G5) was purchased from Zhaowei Electromechanical Co. Ltd, and controlled the rotational degrees of freedom; its speed adjustment range was 0–20 RPM with a rotation angle resolution of 0.05°.

The double-sided tape and PMMA plate were manufactured by a computer numerical control (CNC) laser processing machine (R60 series) according to the model as shown in Fig. 1 ▸(*d*). After that, the Mylar film and processed double-sided tape as well as the processed PMMA plate were assembled according to the steps shown in Fig. 1 ▸(*c*). The ‘connector’ was 3D printed by a Makerbot Replicator 2 device according to the 3D model as shown in Fig. S1 of the supporting information, and then the motor and magnetic base were connected through the ‘connector’.

### 2.3. Crystallization

Lysozyme purified by our laboratory was used for *in situ* crystallization following the microbatch method. The lysozyme was dissolved in ultrapure water to obtain a 40 mg ml^−1^ protein solution, and NaCl was dissolved in ultrapure water at a concentration of 50 mg ml^−1^ to obtain a precipitant solution. Then, the protein solution and the precipitant solution were mixed in equal volumes to obtain the crystallization solution. After that, the crystallization solution was centrifuged at 10000 RPM for 1 min. Then, 5 µL of the supernatant of the crystallization solution was pipetted into the microfluidic channel, and the crystallization solution was guided to the entire microfluidic channel through the pipette tip so that the crystallization solution uniformly nucleated in the microfluidic channel. Finally, the upper unit covered the lower unit containing the crystallization solution to seal the microfluidic channel, and the microfluidic sample plate was placed in an incubator at 20°C for 36 h.

### 2.4. Data collection of the SX experiment

The SX experiment was carried out on BL18U1 at the SSRF equipped with a PILATUS3 X 6M detector. Regarding data collection, the X-ray photon energy was 12.6 keV with a full width at half-maximum (FWHM) of 30 µm × 30 µm (H × V) and a beam flux of 5 × 10^11^ photons s^−1^. The exposure time for each detector frame was 0.3 s. The data acquisition rate was 2 Hz (0.5 s per frame) because the data acquisition included the time to open and close the shutter. The dose rate calculated by *RADDOSE-3D* was 20 kGy s^−1^ (Bury *et al.*, 2018 ▸; Dickerson & Garman, 2021 ▸).

Regarding the 3-DOF-MRT device, the rotating speed (*r*_1_) was 1/6 RPM (the corresponding linear velocity range was 96.0–113.4 µm s^−1^ because the inner and outer radii of the microfluidic channel were 5.5 mm and 6.5 mm, respectively), the step length was 100 µm (with seven steps overall), and the rotational angle (*r*_2_) was 1° per time from −4° to 3° (as determined by a goniometer). More details are listed in Table S1. Before data collection, the microfluidic channel was focused through the control interface of the beamline station.

### 2.5. SX experiment data processing and structural solution

First, the SX data sets were processed through the *CrystFEL* (version 0.8.0) program, including indexing the data using *indexamajig*, scaling using *partialator*, calculating the figures of merit by the *compare_hkl* (*R*_split_, CC_1/2_ and CC*) and *check_hkl* [signal-to-noise ration (SNR), multiplicity and completeness] programs, and exporting the data to *XSCALE* (Kabsch, 2010 ▸) through the *create-xscale* script for further structural refinement (White, 2019 ▸; White *et al.*, 2012 ▸, 2016 ▸).

Then, *Phenix* (version 1.14–3260) was used for molecular replacement (Adams *et al.*, 2010 ▸; Liebschner *et al.*, 2019 ▸) and refinement with 193L as a model (Vaney *et al.*, 1996 ▸). *Coot* (version 0.8.2) was used to manually correct the protein structure (Emsley *et al.*, 2010 ▸). *CCP4* (version 7.0) was used to calculate the 2*F*_o_−*F*_c_ and *F*_o_−*F*_c_ electron density maps (Winn *et al.*, 2011 ▸). Finally, the structure and electron density maps were imaged by *PyMOL* (version 2.2.0) (https://www.pymol.org).

## 3. Results

### 3.1. Effect of rotation on crystal distribution

Lysozyme crystals with an average size of approximately 100 µm × 100 µm × 100 µm and an average distribution density of approximately 15 crystals mm^−2^ in the microfluidic channel were obtained through the *in situ* microbatch method. During sample delivery, the stable position and uniform distribution of crystals in the microfluidic channel were critical. The movement of crystals in the device caused by sedimentation and centrifugal force may interfere with the diffraction pattern, spot size and dimensions, and even data quality. To test the influence of the adopted motion control strategy on the distribution of crystals in the microfluidic channel, we carried out different rotation speed tests (1/6 RPM, 1/5 RPM, 1/4 RPM, 1/3 RPM, 1/2 RPM, 1 RPM, 2 RPM, 4 RPM, 6 RPM, 12 RPM and 20 RPM) and performed statistical analysis on the distribution of crystals. The corresponding centrifugal acceleration range of the crystals was approximately 1.67 µm s^−2^ (1/6 RPM) to 28 µm s^−2^ (20 RPM). In this test, each speed was used to make five full rotations, and three sets of repeated tests were performed. After the rotations were completed, the same position was selected to take a picture and count the percentage of crystals remaining at the initial position.

**Figure 3:**
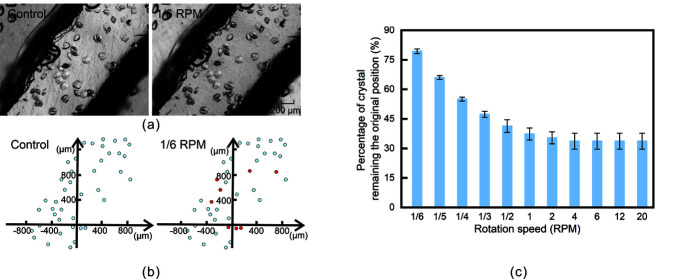
Analysis of crystal distribution. (*a*) Optical imaging of the crystal morphology and its distribution in the microfluidic channel before and after rotation at a speed of 1/6 RPM. (*b*) Schematic diagram of crystal positioning. (*c*) Percentage of crystals remaining at the initial position.

The distribution of crystals in the microfluidic channel before and after rotation at a speed of 1/6 RPM is shown in Fig. 3 ▸(*a*). We located the position of the crystal, as shown in Fig. 3 ▸(*b*). The blue dots indicate that the crystals remained in their initial position, and the red dots mean that the crystals deviated from their initial position. The percentage of crystals remaining at the initial position is shown in Fig. 3 ▸(*c*). The crystal position was stable at low speeds. As the rotation speed increased, the percentage of crystals that remained at the initial position gradually decreased. When the rotation speed reached 4 RPM, the percentage of crystals remaining at the initial position no longer changed with the increase in rotation speed. The crystal could not be maintained at the initial position because the centrifugal force and gravity of crystals, as well as the friction between the crystals and inner wall of the device, could not stay balanced during the movement, as analyzed in Fig. S2 and Section S1 of the supporting information.

In this study, 1/6 RPM was selected as the experimental speed to adapt multiangle SSX research. Under these conditions, approximately 79% of the crystals stably remained at their initial position, and no obvious crystal aggregation occurred. If a faster rotation speed or delivery speed is required, the force of the crystal needs to be considered comprehensively to avoid the influence of crystal aggregation on data analysis. An ideal solution may be to constrain the crystal distribution by referring to the strategy of the sample holder (Baxter *et al.*, 2016 ▸; Illava *et al.*, 2021 ▸; Nam *et al.*, 2021 ▸).

### 3.2. Analysis of background scattering

**Figure 4:**
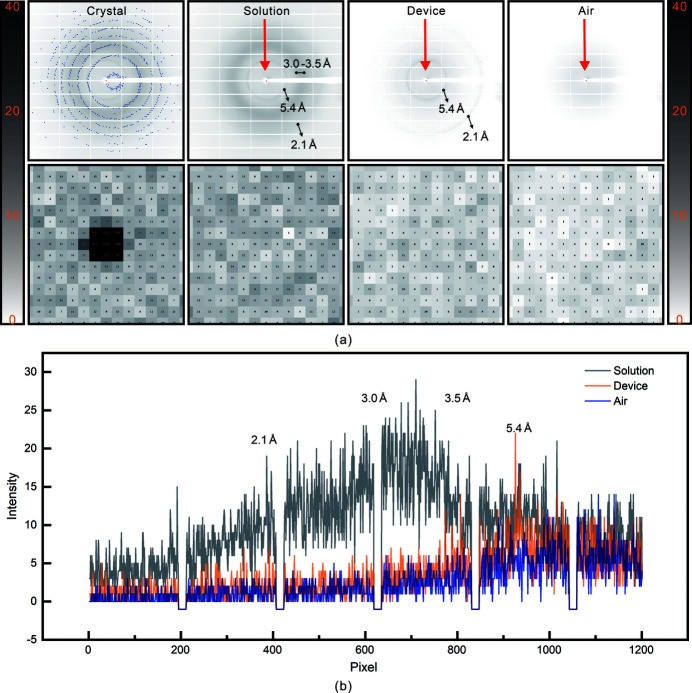
Background scattering analysis. (*a*) Representative diffraction frames and their corresponding magnifications of the crystal, solution, device and air samples. (*b*) Radial profiles of the background scattering intensities of the solution (gray line), device (orange line) and air (blue line) (0 to 1200 pixels along the *y*-axis of the frame as indicated by the red arrow).

As one of the commonly used X-ray transparent films, Mylar film exhibits excellent X-ray transmittance, ultralow X-ray absorption and very weak background scattering. More importantly, it is a chemically inert material that will not affect the physical and chemical environment for crystal growth (Broecker *et al.*, 2016 ▸, 2018 ▸). Considering these advantages, we chose Mylar film with a thickness of 3.6 µm as the diffraction window material for this research, and its X-ray transmittance exceeded 99.9% at an energy level of 12.6 keV (Zhao *et al.*, 2020 ▸). Background scattering analysis was carried out using *ADXV* (version 1.9.14). Fig. 4 ▸(*a*) shows the representative diffraction frames and their corresponding magnifications of the crystal, solution, device and air samples, respectively. Fig. 4 ▸(*b*) shows the radial profiles of the background scattering intensities of the solution (gray line), device (orange line) and air (blue line) from 0 to 1200 pixels along the *y*-axis of the frame, which is indicated by the red arrow in the diffraction frame.

The crystal diffraction frame was derived from the *in situ* grown crystal in the microfluidic channel, which meant that the diffraction intensity came from the crystal, two layers of Mylar film and some crystallization solution around the crystal. The diffraction peak from the crystal was strong enough, although there was some background scattering from the Mylar film and crystallization solution. Then, we analyzed the background scattering of the Mylar film (device) and solution (solution). As a control, we also analyzed the background scattering of air (air). The diffraction frame and radial profile of the device showed weak and thin scattering signals at 5.4 Å and 2.1 Å. The diffraction frame and radial profile of the solution showed another scattering signal between 3.0 and 3.5 Å. The scattering signal generated from the solution is stronger, while, compared with the strong peak of crystal, the scattering signal generated by the solution did not affect the data analysis.

### 3.3. Data collection analysis and structural refinement

#### 3.3.1. Data collection analysis

The SX experiment was carried out on BL18U1 at the SSRF, and 44459 diffraction images from one microfluidic sample plate were collected in approximately 6.5 h, resulting in a sample consumption of 5 µL of crystallization solution or 0.1 mg of protein. Details of the data collection analysis are listed in Table 1 ▸. The *indexamajig* program of *CrystFEL* was used to index all the obtained diffraction data, resulting in an effective indexed diffraction frame of 4470 with a corresponding indexable rate of 10.73%. The average distribution density of crystals in the microfluidic channel is approximately 15 crystals mm^−2^, resulting in only approximately 15% of the microfluidic channel being covered by crystals. The index rate of 10.73% indicates that most crystals can be hit and obtain efficiently indexable diffraction frames. After that, the *partialator* program was used for scaling. The figures of merit were calculated through the *compare_hkl* and *check_hkl* programs, yielding *R*_split_, CC_1/2_, CC* and SNR values of 27.52% (53.41%), 88.47% (57.70%), 96.89% (85.54%) and 3.98 (2.45), respectively.

**Figure 5:**
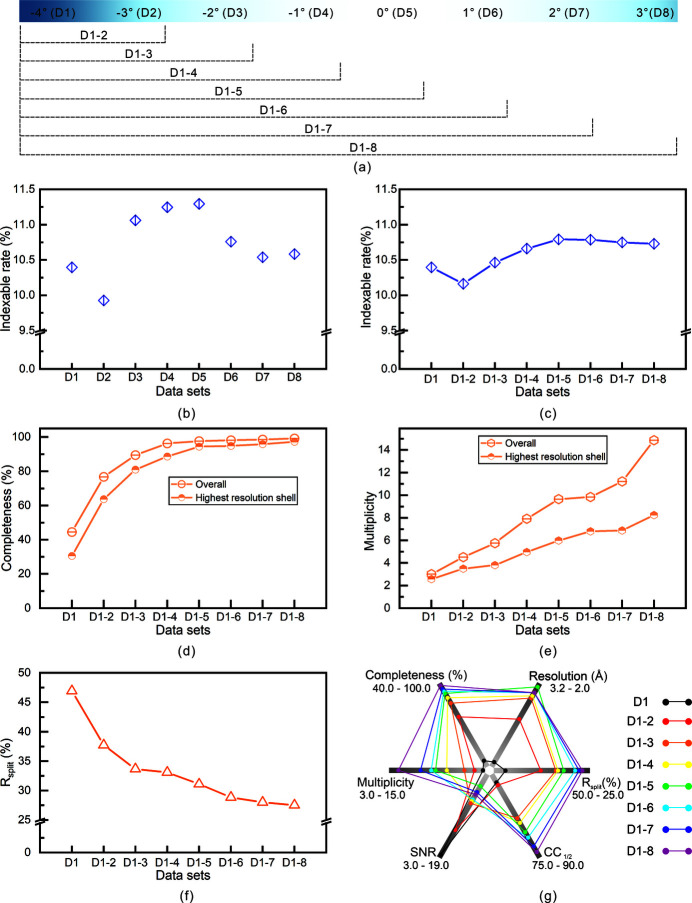
Data collection analysis. (*a*) Strategy of subset grouping. (*b*) Indexable rate of different subsets. (*c*) Indexable rate changes as the diffraction data accumulated. Changes in the figures of merit: (*d*) completeness, (*e*) multiplicity and (*f*) *R*_split_ of the different subsets as the diffraction data accumulated. (*g*) Radar graph of the different subsets plotted by the different figures of merit. The scale of the axis is from the inside to the outside.

To monitor the changes in the diffraction data during the experiment, we performed data collection analysis on different subsets that were grouped as shown in Fig. 5 ▸(*a*). The data collection analysis of different subsets was performed following the above steps. Fig. 5 ▸(*b*) and Table S2 show the indexable rates of the different subsets. The indexable rate was the highest when the rotation (*r*_2_) angle was 0°, that is, when the X-ray beam was orthogonal to the microfluidic sample plate. The statistics of the indexable rate during the rotation process are shown in Fig. 5 ▸(*c*), and the trend derives from the accumulation of diffraction data. These results regarding the indexable rate indicate that the implementation of the rotation degree of freedom (*r*_2_) may cause a slight decrease in the indexable rate, which may result from the misalignment of some crystals after rotation.

Figs. 5 ▸(*d*)–5(*f*) present the change in the figures of merit (completeness, multiplicity and *R*_split_) with the accumulation of diffraction data. More details of the data collection analysis are reported in Table S3. The completeness and multiplicity exhibited obvious increases with the accumulation of diffraction data. *R*_split_ exhibits a better trend with the accumulation of diffraction data. We drew a radar graph to visually exhibit the changes in the different figures of merit. It can be understood that the larger the graph area composed of different figures of merit, the better the data quality. Notably, the subset of D1-5 had a resolution of 2.05 Å, while the resolution of D1-6, D1-7 and D1-8 was 2.15 Å, which may indicate that the quality of the crystals deteriorated during the data collection process. In addition, the SNRs of subsets D1 and D1-2 are higher. This result comes from the fact that their cutoff resolution is low because the data completeness of the high-resolution shell is insufficient. Of course, the result also comes from the fact that the signals are from relatively fresh crystals.

#### 3.3.2. Structural refinement

**Figure 6:**
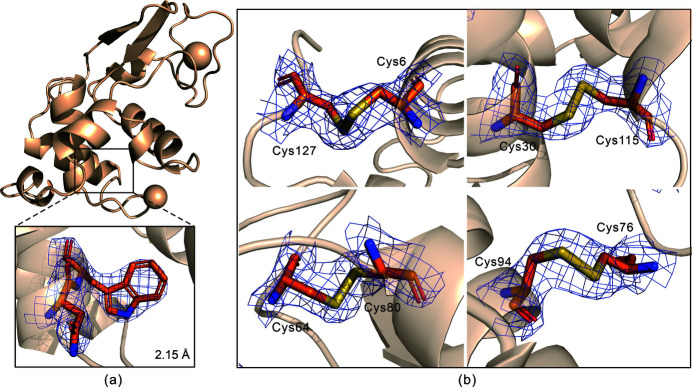
Structural analysis. (*a*) Lysozyme structure and the enlargement of typical residues as well as their electron density maps obtained through the data set of D1-8. (*b*) 2*F*_o_−*F*_c_ (blue mesh, 1.5σ) and *F*_o_−*F*_c_ (red mesh, −3.0σ) electron density maps of the di­sulfide bond in lysozyme.

Structural refinement was performed using *Phenix*. The PDB entry 193L was selected as a model for molecular replacement and refinement. After that, the protein structure was manually corrected using *Coot*. Finally, the resolution of lysozyme was processed to 2.15 Å with a final *R*_work_/*R*_free_ of 18.74%/20.00%. The refined structure was deposited in the PDB database with the code 7dln. The average *B* value of our data is 77.3 Å^2^. Typically, SX obtains a higher *B* value than traditional single-crystal room-temperature diffraction due to the intensity merging method in SX (Stellato *et al.*, 2014 ▸). More details of the structural refinement are listed in Table 1 ▸. Fig. 6 ▸(*a*) shows the lysozyme structure and the enlargement of typical residues as well as their electron density maps. We also analyzed the structure from the D1-3, D1-4, D1-5, D1-6 and D1-7 subsets. The detailed structural information is reported in Table S4. The corresponding structures and electron density maps are presented in Fig. S3.

### 3.4. Radiation damage analysis

Radiation damage, as one of the largest obstacles to obtaining the precise structure of biological macromolecules at room temperature, has been greatly alleviated since the advent of SX. We performed Fourier difference maps calculations of di­sulfide bonds to evaluate the radiation damage.

There are four di­sulfide bonds in lysozyme (Cys6—Cys127, Cys30—Cys115, Cys64—Cys80 and Cys76—Cys94) that are susceptible to radiation damage. The *FFT* program of *CCP4* (version 7.0) was used to calculate the 2*F*_o_−*F*_c_ and *F*_o_−*F*_c_ electron density maps. Then, the 2*F*_o_−*F*_c_ and *F*_o_−*F*_c_ electron density maps of di­sulfide bonds were imaged by *PyMOL* (version 2.2.0), as shown in Fig. 6 ▸(*b*). Regarding the analyzed structures in this study, no obvious *F*_o_−*F*_c_ electron density was found at a contour level of −3.0σ. This result indicated that the lysozyme structure obtained by our proposed device and method did not suffer serious radiation damage. However, an increase in the rotation (*r*_2_) range will inevitably increase the radiation damage. Thus, it is necessary to consider the cumulative effect of the radiation dose to avoid or reduce radiation damage. In addition, radicals will be generated by radiation damage by exposing crystals continuously. Therefore, the proposed method should be applied to samples sensitive to radiation damage with caution.

## 4. Discussion

### 4.1. Comparison of 3-DOF-MRT and 2-DOF-MRT

Compared with our previous SX study on the 2-DOF-MRT device (Zhao *et al.*, 2020 ▸), this 3-DOF-MRT device exhibits a significantly reduced sample consumption and shortened time requirement; moreover, the indexable rate clearly increases, as reported in Table S5. These results benefit from the 3-DOF motion control strategy, which enables the full application of crystals. In addition, adequate adaptation of the 3-DOF-MRT device to the diffraction environment contributes to obtaining diffraction frames that are easier to process by software. For example, there is no strong diffraction ring that comes from the platinum aperture in the study of the 3-DOF-MRT device, while that is observed in the study of the 2-DOF-MRT device. In the study of the 2-DOF-MRT, the capillary in the diffraction facility is lowered to make enough space, which leads to strong diffraction rings coming from the platinum aperture.

### 4.2. Analysis of 3-DOF-MRT device performance

The rotation speed range of the motor used in the 3-DOF-MRT device is 0–20 RPM, which means that the corresponding sample delivery speed range was approximately 0–1.36 × 10^−2^ m s^−1^ because the outer radius of the microfluidic channel is 6.5 mm. Theoretically, the range of the rotational speed can be from 0 to very large values, depending on the capability of the hardware. However, we need to consider the centrifugal acceleration that different samples can withstand, depending on the features of different protein crystals.

Although the 3-DOF-MRT device has made great progress compared with the 2-DOF-MRT device, collecting a full dataset in 6.5 h is extremely long, both for single cryo-frozen crystallography and for SX. However, the effective time to acquire a complete dataset can be drastically improved at a microfocus beamline with a faster detector. For example, if a data collection rate of 100 Hz (0.01 s frame^−1^) is applied, the data collection time can be reduced to less than 10 min.

### 4.3. Strategy of motion control parameters selection

**Figure 7:**
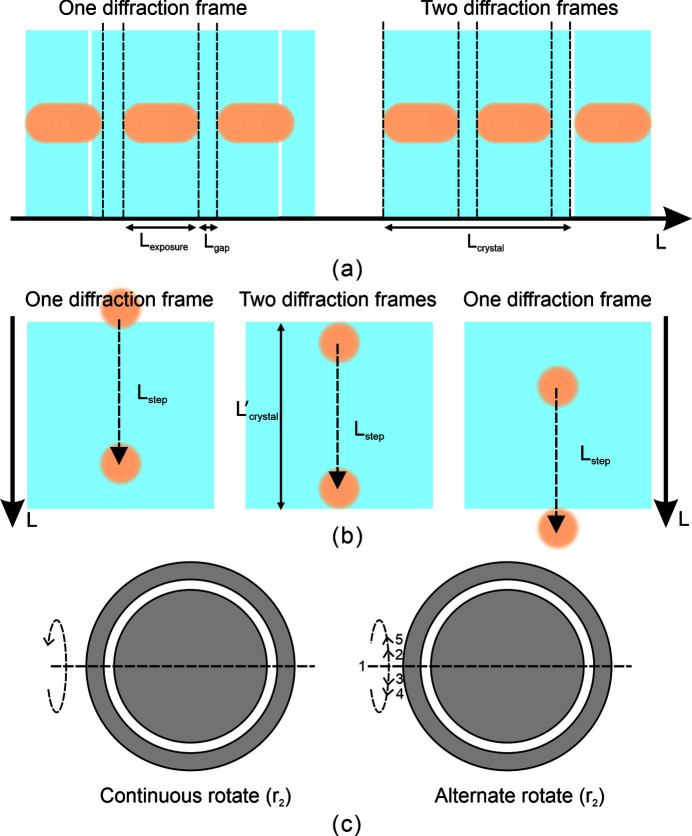
Recommended sample delivery speed, step length and rotational (*r*_2_) mode. (*a*) Recommended sample delivery speed. *L*_crystal_ is the size of the crystal in the delivery direction, *L*_exposure_ is the distance of the crystal delivery during exposure, and *L*_gap_ is the distance of the crystal delivery during the delay time for the detector collecting data. (*b*) Recommended step length. 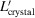 is the size of the crystal in the translation direction, and *L*_step_ is the translation step length. (*c*) Recommended rotational (*r*_2_) modes, including continuous rotation in one direction and alternating rotation in two directions.

Sample delivery technology based on circular motion is worthy of promotion. The selection of motion control parameters [rotation (*r*_1_) speed, translation step length and rotation (*r*_2_) mode] is discussed to provide a reference. The rotation speed corresponds to the sample delivery speed. If the X-ray beam contacts the crystal completely during a single exposure, this contact is regarded as effective diffraction. The model presented below would be an ideal choice for the sample delivery speed,

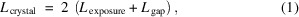




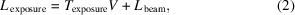

where *L*_crystal_ is the size of the crystal in the delivery direction, *L*_exposure_ is the distance of the crystal delivery during exposure, *L*_gap_ is the distance of the crystal delivery during the delay time for the detector collecting data, *T*_exposure_ is the exposure time, *V* is the sample delivery speed, and *L*_beam_ is the size of the X-ray beam. Under these conditions, each crystal can collect at least one and up to two diffraction frames, as shown in Fig. 7 ▸(*a*).

The model presented below is an ideal choice for the translation step length,

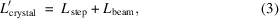

where 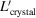 is the size of the crystal in the translation direction and *L*_step_ is the translation step length. Under the recommended translation step length, each crystal can collect at least one and up to two diffraction frames, as shown in Fig. 7 ▸(*b*). Since the shape of different crystals varies, the crystal size in the delivery direction and translation direction is not intuitive. It will be more instructive to use the size of the inscribed square as the size of the crystal in the delivery and translation directions. To more conveniently use this method, the length of the short side of the crystal can be directly used as the crystal size in the delivery direction and translation direction.

In this study, the rotation (*r*_2_) of the crystal is continuously performed in one direction. Relevant experimental data show that the highest indexable rate will be obtained when the X-ray beam is orthogonal to the microfluidic sample plate; thus, the rotation range should not be too large. Therefore, we demonstrate another reference rotation mode, that is, alternate rotation, as shown in Fig. 7 ▸(*c*). Starting from the middle, follow the number signs to alternately swing in two directions, which may help to obtain better diffraction results. In addition, for smaller crystals, there is a typical problem of crystal settling, which can be weakened by alternate rotation.

## 5. Conclusions

Sample delivery based on circular motion has become a valuable mode because its delivery speed can be adjusted flexibly and made applicable to many different platforms; therefore, it is worthy of further development to fully utilize the advantages. In this paper, oscillating serial crystallography and circular-motion-based serial crystallography have been combined, and a novel microfluidic rotating-target device capable of three-degrees-of-freedom motion has been developed. By collecting multiple diffraction frames from each crystal in the microfluidic channel, the sample consumption can be greatly reduced, and the efficiency of sample delivery can be highly improved. In addition, the new device retains the technical advantages of *in situ* diffraction and room-temperature structural determination. Furthermore, the device is easy to manufacture and can be conveniently implemented on synchrotron radiation facilities. Due to these advantages, we strongly recommend the practical utilization of this technology in SSX.

## Supplementary Material

mmcif file. DOI: 10.1107/S1600577523000462/wz5028sup1.mcf

Structure factors: contains datablock(s) r7dlnsf. DOI: 10.1107/S1600577523000462/wz5028sup2.hkl

Movie S1. An animation demonstration of the motion control scheme. DOI: 10.1107/S1600577523000462/wz5028sup3.avi

Supporting Figures S1 to S3; Tables S1 to S5. DOI: 10.1107/S1600577523000462/wz5028sup4.pdf

## Figures and Tables

**Table 1:** Data collection analysis and structural refinement for lysozyme (D1-8)

Crystal size (µm)	100 × 100 × 100
Exposure time (s)	0.3
Wavelength (Å)	0.98
Photon energy (keV)	12.6
FWHM (H × V) (µm)	30 × 30
Beam flux (photons s^−1^)	5 ×10^11^
No. of indexed / collected images / indexable rate	4770 / 44459 / 10.73%
Detector distance (mm)	300
Resolution (Å)	34.24–2.15 (2.23–2.15)
Space group	*P*4_3_2_1_2
*a*, *b*, *c* (Å) α, β, γ (°)	79.8, 79.8, 38.7 90, 90, 90
*R*_split_ (%)	27.52 (53.41)
CC_1/2_ (%)	88.47 (57.70)
CC* (%)	96.89 (85.54)
*I*/σ(*I*) (SNR)	3.98 (2.45)
Completeness (%)	99.26 (97.20)
*R*_work_ / *R*_free_ (%)	18.74 / 20.00
Average *B* value (Å^2^)	77.3
Total number of reflections	6887
Number of reflections in refinement	6200
Number of free reflections in refinement	687
Number of atoms	1050
Protein	1000
Water and others	50
R.m.s deviations from ideal values	
Bonds (Å)	0.0092
Angles (°)	1.15
Ramachandran plot statistics (%)	
Favored	96.06
Allowed	3.94
Disallowed	0
PDB entry	7dln
