# Supplementary material for: Microfluidic rotating-target device capable of three-degrees-of-freedom motion for efficient in situ serial synchrotron crystallography
Source: J Synchrotron Radiat. 2023 Feb 15;30(Pt 2):347–58. doi: 10.1107/S1600577523000462 (PMC10000801; doi:10.1107/S1600577523000462)
Supplement: Supplementary file 4 [file s-30-00347-sup4.pdf]

JOURNAL OF SYNCHROTRON RADIATION

Volume 30 (2023)

Supporting information for article:

Microfluidic rotating-target device capable of three-degrees-of-freedom motion for efficient *in situ* serial synchrotron crystallography

Feng-Zhu Zhao, Zhi-Jun Wang, Qing-Jie Xiao, Li Yu, Bo Sun, Qian Hou, Liang-Liang Chen, Huan Liang, Hai Wu, Wei-Hong Guo, Jian-Hua He, Qi-Sheng Wang and Da-Chuan Yin

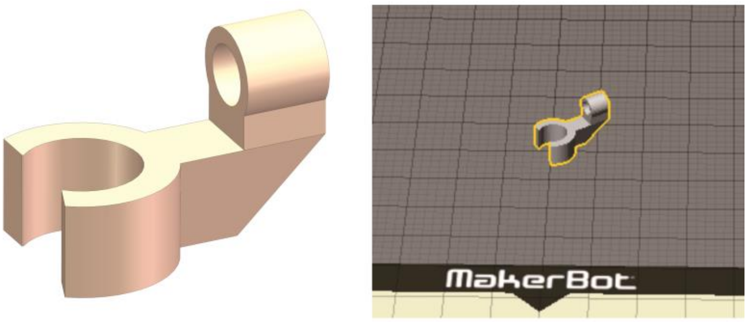

(a) (b)

Figure S1 Diagram of the "connector". (a) 3D model of the "connector". (b) The "connector" is manufactured by 3D printing.

Table S1 Parameters of the 3-DOF-MRT device

		Parameters
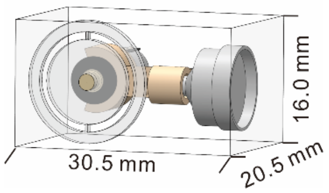	Volume	30.5 ×20.5 ×16 mm^3
	Inner radius of the microfluidic channel	5.5 mm
	Outer radius of the microfluidic channel	6.5 mm
	Rotational speed (r_1)	1/6 RPM
	Sample delivery speed	96.0-113.4 μm/s
	Rotational angle (r_2)	1°/times (-4° to 3°)
	Step length	100 μm/times
	Rotational speed adjustment range of the motor	0-20 RPM
	Sample delivery speed adjustment range	0-1.36×10^{-2} m/s

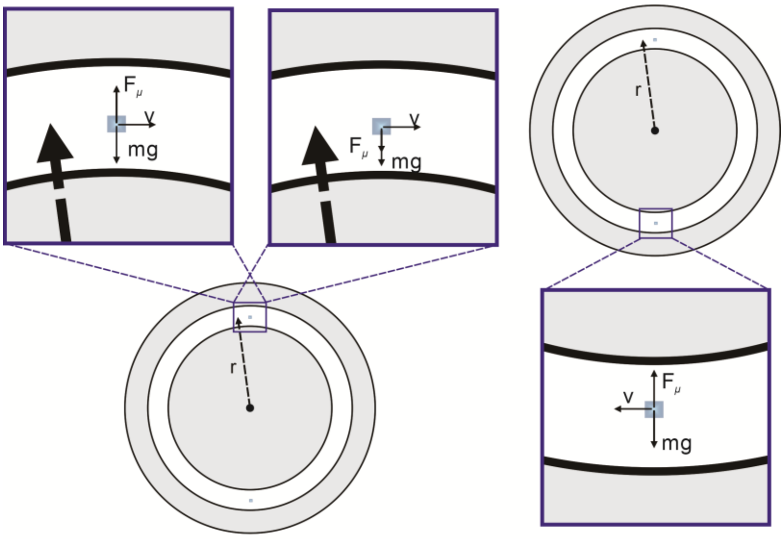

Figure S2 Force analysis diagram of the crystal at the top (a) and bottom (b) of the microfluidic channel.

S1. Supplementary Discussion

According to the force analysis, the following circumstances may cause the crystals to deviate from their initial position.

At the top of the microfluidic channel,

$$\frac{mv^2}{r} \geq \mathrm{mg} - F_{\mu max} \qquad (1)$$

$$\frac{mv^2}{r} \leq \mathrm{mg} + F_{\mu max} \qquad (2)$$

At the bottom of the microfluidic channel,

$$\frac{mv^2}{r} \leq F_{\mu max} - \mathrm{mg} \qquad (3)$$

Under the condition of (1), the maximum static friction force ($F_{\mu max}$) of the crystal is not enough to resist gravity (mg) and centripetal force ($\frac{mv^2}{r}$), resulting in the crystal sliding inward. Under the condition of (2), the maximum static friction force and gravity are in the same direction, and the maximum static friction force and gravity are not enough to provide centripetal force, thereby resulting in the crystal moving outward. Under the condition of (3), the maximum static friction force is not enough to resist gravity and centripetal force, resulting in the crystal moving outward.

Table S2 Indexable rate of the diffraction data set obtained under different rotation(r_2) angles.

	D1 (-4 °)	D2 (-3 °)	D3 (-2 °)	D4 (-1 °)	D5 (0 °)	D6 (1 °)	D7 (2 °)	D8 (3 °)
No. indexed /collected images	572 / 5502	542 / 5459	610 / 5514	628 / 5584	636 / 5631	599 / 5568	591 / 5608	592 / 5593
Indexable rate [%]	10.40	9.93	11.06	11.25	11.29	10.76	10.54	10.58

Table S3 Data collection statistics.

	D1	D1-2	D1-3	D1-4	D1-5	D1-6	D1-7	D1-8
No. indexed /collected images	572 / 5502	1114 / 10961	1724 / 16475	2352 / 22059	2988 / 27690	3587 / 33258	4178 / 38866	4770 / 44459
Indexable rate [%]	10.40	10.16	10.46	10.66	10.79	10.79	10.75	10.73
No. used images [a)]	375	828	1285	1605	1857	1992	2210	2244
Space group	P43212	P43212	P43212	P43212	P43212	P43212	P43212	P43212
a, b, c [Å]	79.8, 79.8, 38.7	79.8, 79.8, 38.7	79.8, 79.8, 38.7	79.8, 79.8, 38.7	79.8, 79.8, 38.7	79.8, 79.8, 38.7	79.8, 79.8, 38.7	79.8, 79.8, 38.7
α, β, γ [°]	90, 90, 90	90, 90, 90	90, 90, 90	90, 90, 90	90, 90, 90	90, 90, 90	90, 90, 90	90, 90, 90
Resolution [Å] [b)]	3.10	2.48	2.20	2.18	2.05	2.15	2.15	2.15
SNR	18.75 (18.18)	13.54 (5.34)	4.48 (4.00)	4.36 (3.26)	3.56 (2.32)	4.20 (2.42)	3.85 (2.87)	3.98 (2.45)
R_{split} [%]	46.92 (42.00)	37.75 (56.35)	33.68 (49.02)	33.11 (57.52)	31.09 (56.63)	28.83 (55.01)	27.99 (48.89)	27.52 (53.41)
$CC_{1/2}$ [%]	76.41 (56.93)	77.02 (52.90)	82.85 (63.66)	83.40 (52.72)	85.53 (58.71)	86.59 (57.75)	88.57 (64.42)	88.47 (57.70)
CC^* [%]	93.07 (75.17)	93.29 (83.19)	95.20 (88.20)	95.37 (83.09)	96.02 (86.01)	96.34 (85.57)	96.92 (88.52)	96.89 (85.54)
Completeness [%]	44.53 (30.64)	76.79 (63.81)	89.51 (80.98)	96.28 (88.65)	97.70 (94.48)	98.14 (94.84)	98.63 (95.93)	99.26 (97.20)
Multiplicity	3.02 (2.59)	4.53 (3.50)	5.76 (3.82)	7.92 (4.97)	9.66 (5.99)	9.85 (6.81)	11.22 (6.89)	14.17 (8.25)

[a)] No. of images used for scaling.

[b)] The resolution cutoff is based on $CC_{1/2}$.

Table S4 Structural refinement statistics.

	D1-2	D1-3	D1-4	D1-5	D1-6	D1-7	D1-8
Resolution [Å]	35.33 - 2.48	35.33 - 2.20	35.33 - 2.18	35.33 - 2.05	34.24 - 2.15	31.42 - 2.15	34.24 - 2.15
	(2.56 - 2.48)	(2.28 - 2.20)	(2.26 - 2.18)	(2.12 - 2.05)	(2.23 - 2.15)	(2.23 - 2.15)	(2.23 - 2.15)
Total No. of reflections	4009	6204	6545	7835	6849	6867	6887
No. of reflections in refinement	3611	5584	5890	7047	6165	6178	6200
No. of free reflections in refinement	398	620	655	788	684	689	687
R_{work}/R_{free} [%]	25.05 / 26.79	23.91 / 25.75	21.66 / 22.57	20.87 / 23.35	20.76 / 22.81	19.75 / 22.38	18.74 / 20.00
Average B value [Å^2]	61.7	69.7	72.3	70.0	77.7	79.0	77.3
No. of atoms	1030	1043	1051	1056	1048	1043	1050
Protein	1001	1001	1001	1000	1001	1001	1000
Water and others	29	42	50	56	47	42	50
R.m.s deviations from ideal values							
Bonds [Å]	0.0092	0.0068	0.0076	0.0064	0.0078	0.0082	0.0092
Angles [°]	1.10	0.92	0.99	0.87	1.03	1.07	1.15
Ramachandran plot statistics [%]							
Favored	95.28	96.06	98.43	97.64	97.64	98.43	96.06

Allowed	4.72	3.94	1.57	3.36	3.36	1.57	3.94
Disallowed	0	0	0	0	0	0	0
PDB entry	---	---	---	---	---	---	7DLN

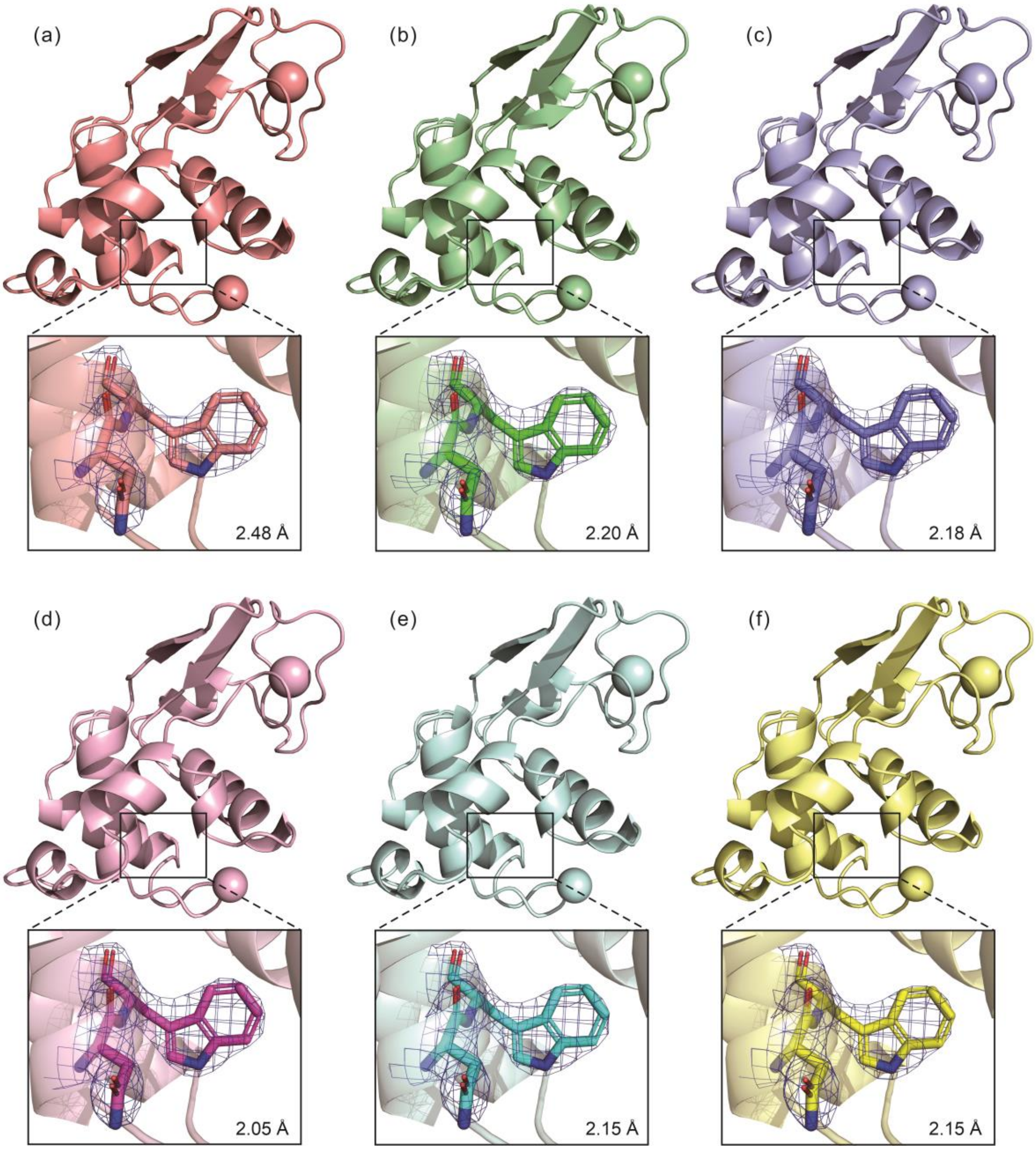

Figure S3 Lysozyme structures and the enlargement of typical residues as well as their electron density maps obtained through the following subsets: (a) D1-2, (b) D1-3, (c) D1-4, (d) D1-5, (e) D1-6, and (f) D1-7.

Table S5 Comparison of typical parameters between 2-DOF-MRT device and 3-DOF-MRT device.

	2-DOF-MRT	3-DOF-MRT (D1-8)
Indexable rate (%)	2.28	10.73
Data collection rate (Hz)	2	2
No. of indexable frames collected per hour	137	688
Protein consumption (mg)	4.32	0.1
Total measuring time (h)	18	6.5
